# Exploring the Relationship Between Drivers’ Stationary Gaze Entropy and Situation Awareness in a Level-3 Automation Driving Simulation

**DOI:** 10.1177/10711813241275910

**Published:** 2024-08-29

**Authors:** Wen Ding, Yovela Murzello, Shi Cao, Siby Samuel

**Affiliations:** 1 University of Waterloo, ON, Canada

**Keywords:** stationary gaze entropy, situation awareness, eye tracking, level-3 autonomous driving

## Abstract

The transition period from automation to manual, known as the takeover process, presents challenges for drivers due to the deficiency in collecting requisite contextual information. The current study collected drivers’ eye movement in a simulated takeover experiment, and their Situation Awareness (SA) was assessed using the Situation Awareness Global Assessment Technique (SAGAT) method. The drivers’ Stationary Gaze Entropy (SGE) was calculated based on the percentages of time they spent on six pre-defined Areas of Interests (AOIs). Three critical time windows were extracted by using the takeover alert time spot and the hazard perceived time spot. The result indicated that drivers with higher SAGAT scores would spread their attention among multiple AOIs. Also, drivers’ SGE and SA have a linear relationship only at the last time window (hazard perceived to the end) wherein SGE potentially functions as an evaluative metric for assessing SA in the future.

## Introduction

### Background

With the progression of technology, level-3 automation cars have become the trend of next-generation self-driving vehicles. While automation offers numerous advantages, it also introduces new risks during the driving process (Azevedo-Sa et al., 2021). The transition period from automated driving systems to manual control, often referred to as the takeover process, introduces significant challenges for drivers. This difficulty arises primarily because drivers often struggle to gather and incorporate the requisite contextual information needed for safe and effective manual control (Cunningham & Regan, 2015). Following a period of not being immersed in maneuvering, drivers may lack information on current vehicle status or appropriate subsequential actions when they are requested to do a takeover. Additionally, in level-3 automated vehicles, drivers are allowed to do non-driving-related tasks, including reading, chatting, or even sleeping (McCall et al., 2016). Previous research has demonstrated that these activities can exacerbate the challenge of conducting a takeover effectively (Naujoks et al., 2018). Drivers’ SA plays a critical role in acquiring sufficient information in the takeover process, in both automation and manual driving. However, monitoring drivers’ SA is challenging because cognitive information processing occurs internally. Since human drivers are a critical component of level-3 autonomous cars, it is essential to evaluate their SA levels using external physiological measures, such as eye tracking (Zhang et al., 2020). Gaze behaviors are closely related to drivers’ cognitive activities. One important metric of gaze behavior is the randomness of eye movements, known as entropy, which measures the variability and distribution of fixations, reflecting how a person’s gaze is distributed across various AOIs.

This study seeks to explore the relationship between drivers’ SGE and their SA in the takeover process in level-3 automation driving conditions.

### Related Works

The concept of SA, first proposed by Endsley (1988a), is defined as the perception of environmental elements within a given time and space, the understanding of their significance, and the prediction of their future status. A substantial body of research has concentrated on enhancing SA in the takeover process, as studies have shown that higher SA levels significantly improve the ability to regain control (Fu et al., 2024; Li et al., 2023; McKerral et al., 2023). Previous research has demonstrated that eye tracking features can evaluate people’s SA effectively. Liang et al. (2021) discovered better overall SA correlates with longer time spent viewing the driving scene and more dispersed visual attention allocation in semi-autonomous driving. Zhou et al. (2022) used the model LightGMB to predict the SA scores with eye tracking features and got great accuracy.

Gaze entropy is a well-developed metric representing people’s gaze behaviors quantitatively. It has gained more and more attention in recent years due to its ability to describe the average information or uncertainty associated with choices (Shannon, 1948; Shiferaw et al., 2019). Previous research has indicated the feasibility of utilizing entropy to assess SA (van de Merwe et al., 2012). Yang et al. (2023) investigate how entropy is correlated with comprehension in situational awareness for autonomous driving. The SGE is a commonly used entropy metric in many previous studies. In this study, the SGE was employed to measure gaze distribution over a specified period. The more equally the fixation is distributed, the higher the SGE, indicating a searching gaze behavior. Thus, a lower SGE indicates a more concentrated gaze behavior (Ayala et al., 2023; Shiferaw et al., 2019).

This paper attempted to build models between the SGE and the SA on different time windows in one takeover process, aiming to explore the possibility of evaluating drivers’ SA using SGE as an eye tracking feature in the future. Developing these models between the SGE and SA represents a significant advancement in the establishment of driver monitoring systems, which have the potential to enhance takeover safety in level-3 automation vehicles.

## Methodology

### Experiment Design

In the current study, a simulated driving experiment was implemented using CARLA, an open-source driving imulation software for autonomous vehicles (Dosovitskiy et al., 2017).

**Figure 1.**
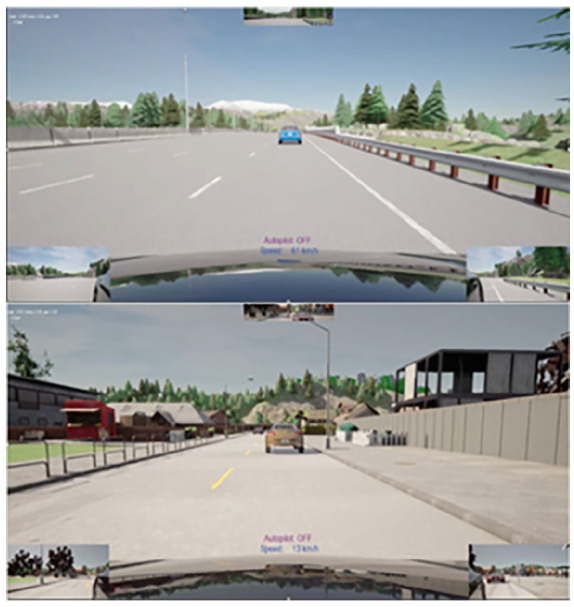
Simulated driving scenarios (up: highway, down: city).

Each participant went through eight driving scenarios with different road types and drive types (see Figure 1), during which their eye movement was collected by Dikablis 3 eye tracking glasses. In each scenario, the participants enabled the autonomous driving function from the beginning. The autonomous system would take them along designated trajectories at 90 km/h on highways and 30 km/h on city roads. When the vehicle reached the specified spot, the autonomous system would give a takeover alert to the human drivers in both visual and auditory formats. In each scenario, a hazard scene would appear a few seconds after the takeover alert. Possible hazard scenes include stopped lead vehicles, collisions, and road debris. The human driver was expected to detect the hazard scene and avoid it. The scenario would end automatically around 50 m after the hazard scene. The driving simulations were displayed on a 27-inch 1080p monitor, with a Logitech G29 steering wheel and pedal set.

### Participants

The participants’ consent was obtained before the commencement of the experiment. They were required to sign the consent form and finish a demographic questionnaire about their driving experience. Since most participants did not have any experience with autonomous vehicles, they were briefed about the experiment content and their role in the level-3 automation driving and takeover process. They were notified that this takeover alert was caused by the incapability of the autonomous system, and they are expected to take over the car with a minimum time delay.

In total, 48 drivers with valid Canadian Driver’s Licenses were involved in the current study (*M* = 31.56; *SD* = 4.13), and the participants included 22 female and 26 male drivers.

## Results

### Data Pre-Processing

#### Area-of-Interests (AOIs)

**Figure 2.**
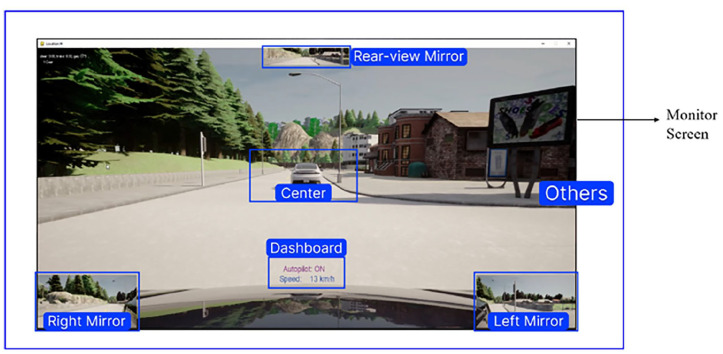
AOIs for this study (the AOI “others” covers all the remaining vision fields).

The AOIs refer to specific regions within the driver’s field of vision that contain task-related information. Researchers determined these AOIs using various criteria such as expert experience, attention maps, or clustering algorithms (Mao et al., 2021). Figure 2 demonstrates the six AOIs defined for the current study. Most AOIs are within the monitor area because most driving information was presented on the monitor screen.

#### Stationary Gaze Entropy (SGE)

The drivers’ SGE was calculated based on the percentages of time they spent on six pre-defined AOIs above, which are the rear mirror, left mirror, right mirror, center of the road, dashboard, and other areas. The gaze location was obtained by implementing a Coordinates affine transfer method based on the markers affixed by the corners of the simulation screen ahead of time (Ding et al., 2023). At last, the SEG was calculated based on the equation proposed by Shannon (1948) as shown below.

$$H(x)=\sum_{i=0}^{n}\left(p_{i}\right){log}_{2}(p_{i})$$

#### SAGAT Scores

In the current experiment, drivers’ SA was assessed by the SAGAT method proposed by Endsley (1988b). After each experimental trial, they will be asked two SAGAT questions which cover the information needed to be collected during the whole driving process. Each response to the SAGAT questions was scored between 0 and 1, with partially correct answers receiving a score of 0.5. Consequently, each participant could receive one of five possible scores ranging from 0 to 2, in increments of 0.5, for each driving trial.

### Data Analysis Results

The SGE was analyzed using three separate time windows. The hazard put after the takeover spot was an important occasion and needed to be perceived as soon as possible. Therefore, the moment that the hazard was perceived becomes an important time spot. Three critical time windows were extracted by using the takeover alert time spot and the hazard perceived time spot: 10 s before the takeover alert, from the takeover alert to the hazard perceived, and from the hazard perceived to the end of the trial.

**Figure 3.**
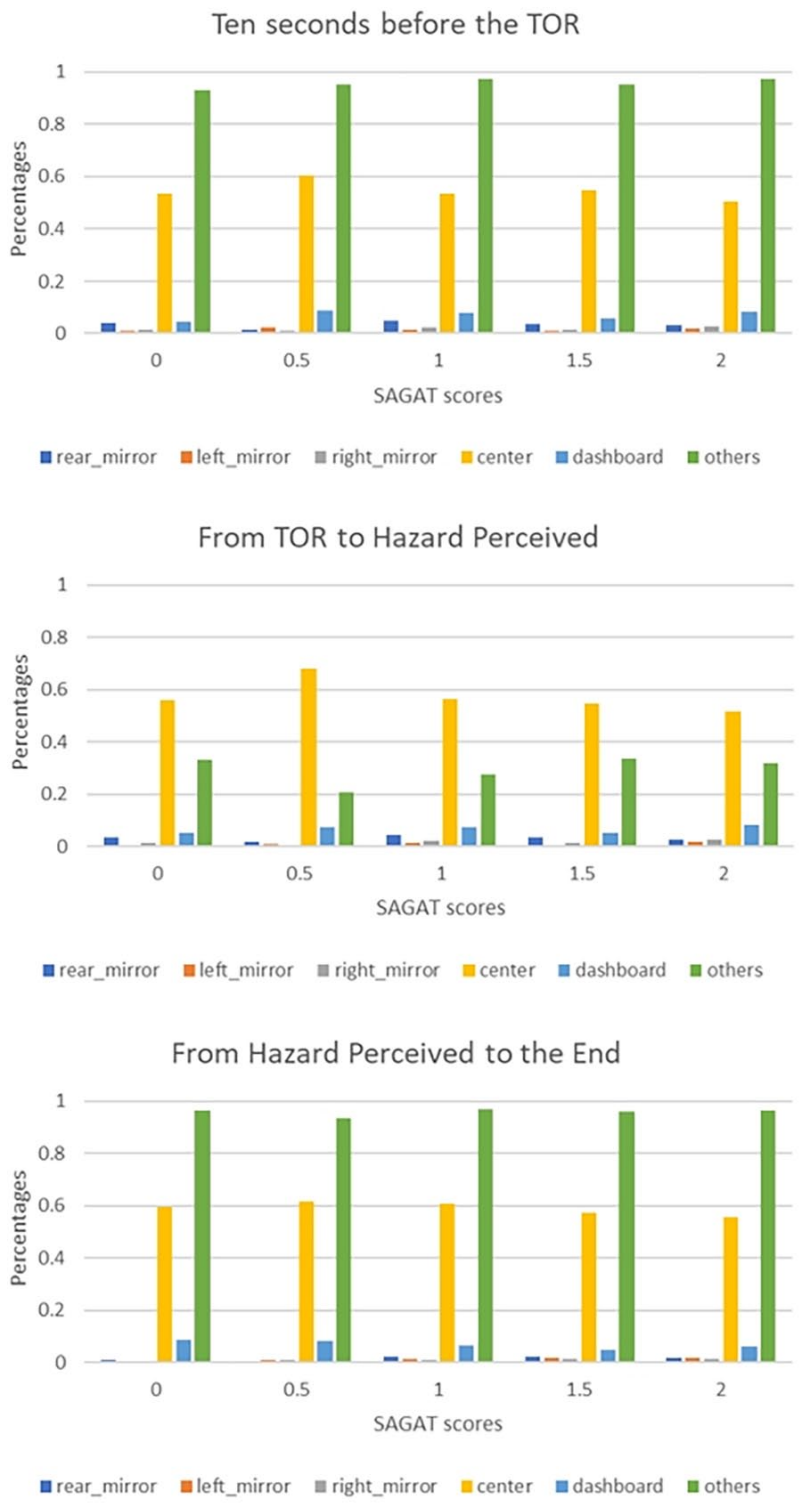
Gaze percentage on six AOIs among five levels SAGAT scores on three time windows.

For each time window, the percentages of time spent in six AOIs were compared among 5 levels of drivers’ SAGAT scores (see Figure 3). The result indicated that drivers with higher SAGAT scores would spread their attention among multiple AOIs. Three figures from top to bottom are three different time windows mentioned above. From the figure, we can tell those trials with 2 scores, which is the highest score a driver can get, distributed their gaze among six AOIs more equally. And this trend is the same for two other time windows. A clearer trend can be seen from the regression analysis next.

**Figure 4.**
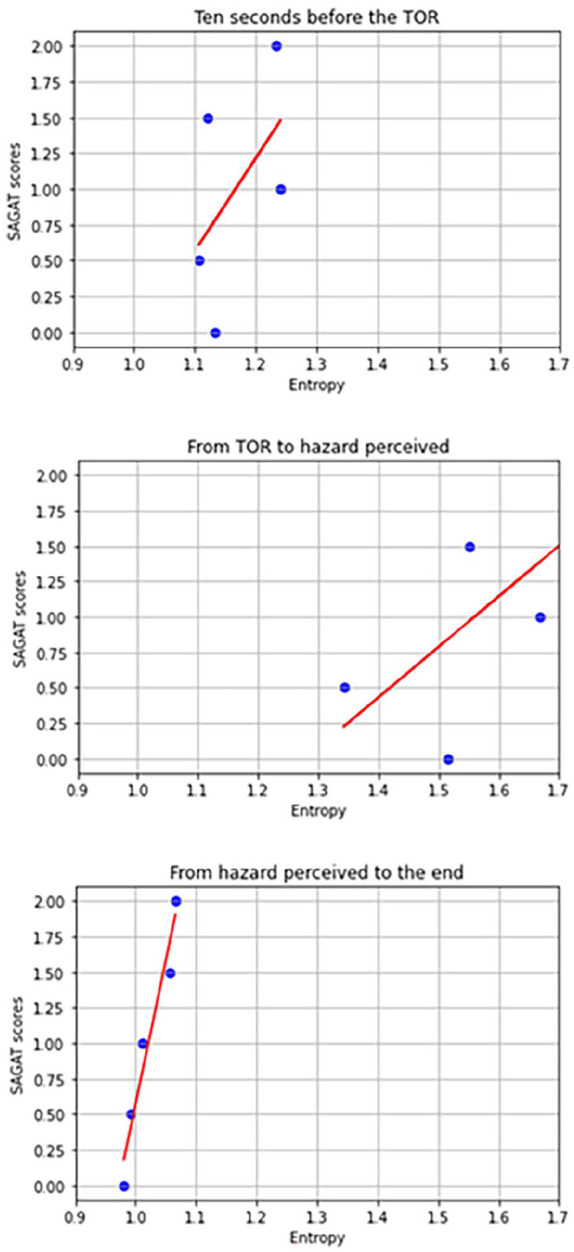
Linear regressions between SGE and SA on three time windows.

Subsequently, three linear models were developed to examine the relationship between drivers’ SGE and SAGAT scores across three different time windows. While all three time windows exhibited varying degrees of linearity, statistical significance was achieved only in the linear model for the final time window (from hazard perceived to the end of the trial). Figure 4 illustrates the linear regression results for these three time windows, using the same range for both the *x* and *y* axes.

From hazard perceived to the end of the trial, the results of the regression indicated that the model explained 94.9% of the variance (*R*^2^ = .949, *F* (1, 3) = 55.31, *p* = .005).

Table 1 summarizes the linear regression results of the time window “from hazard perceived to the end of the trial.” SGE was found to be a significant predictor of SAGAT scores (β = 20.23, *t* (3) = 7.437, *p* = .005). These results suggest that the SGE is positively associated with higher SAGAT scores. The other two linear models did not show significant coefficients.

**Table 1.** Linear Regress Result.

Variable	Coefficient	*SE*	*t*-Value	*p*-Value
Intercept	−19.66	2.779	−7.073	.006
SGE	20.23	2.721	7.437	.005

## Conclusion

These results suggest that SGE and SA have a linear relationship only after the hazard is perceived, wherein SGE potentially functions as an evaluative metric for assessing SA. The SAGAT questions used in the current study cover the whole time span including before and after the takeover, so the SAGAT scores represent drivers’ overall SA performance in all time windows. Usually speaking, drivers are encouraged to gain more information while driving to maintain their SA, necessitating the spreading of their attention among more AOIs, consequently resulting in high entropy values. Nevertheless, such a case is not universally applicable. Considering the fact that drivers are required to detect hazards with a minimum time delay after the takeover, their primary vision focus is the center of the road where hazards and adverse events mainly happen. Therefore, drivers are unlikely to disperse their attention uniformly across all AOIs to gather additional information, which would yield higher entropy levels, nor are they inclined to fixate exclusively on a single AOI, resulting in diminished entropy.

## Discussion

In future work, a larger sample size might be able to elucidate a more definitive relationship between SGE and SA. Additionally, the SAGAT questions used in this study had limitations, as only two questions were asked after each trial. There is still much information related to the three levels of SA that could be incorporated into SAGAT questions. Furthermore, the integration of transition entropy into future investigations is recommended, as SGE solely reflects entropy levels within each trial, whereas transition entropy has the capacity to show the sequential patterns of gaze transitions among distinct AOIs.
